# Relationship between Eating Alone and Poor Appetite Using the Simplified Nutritional Appetite Questionnaire

**DOI:** 10.3390/nu14020337

**Published:** 2022-01-14

**Authors:** Yurie Mikami, Keiko Motokawa, Maki Shirobe, Ayako Edahiro, Yuki Ohara, Masanori Iwasaki, Misato Hayakawa, Yutaka Watanabe, Hiroki Inagaki, Hunkyung Kim, Shoji Shinkai, Shuichi Awata, Hirohiko Hirano

**Affiliations:** 1 Research Team for Promoting Independence and Mental Health, Tokyo Metropolitan Institute of Gerontology, 35-2, Sakae-cho, Itabashi-ku, Tokyo 173-0015, Japan; ega0dm@gmail.com (Y.M.); aedahiro514@gmail.com (A.E.); yohara@tmig.or.jp (Y.O.); iwasaki@tmig.or.jp (M.I.); inagaki@tmig.or.jp (H.I.); kimhk@tmig.or.jp (H.K.); awata@tmig.or.jp (S.A.); h-hiro@gd5.so-net.ne.jp (H.H.); 2 The Tokyo Metropolitan Support Center for Preventative Long-Term and Frail Elderly Care, Tokyo Metropolitan Institute of Gerontology, 3-9-7, Itabashi, Itabashi-ku, Tokyo 173-0004, Japan; mashirobe@gmail.com; 3 Integrated Research Initiative for Living Well with Dementia, Tokyo Metropolitan Institute of Gerontology, 25-8, Oyamakanai-cho, Itabashi-ku, Tokyo 173-0024, Japan; erurinrun0424@gmail.com; 4 Gerodontology, Department of Oral Health Science, Faculty of Dental Medicine, Hokkaido University, Kita 8, Nishi 5, Kita-ku, Sapporo 060-0808, Japan; ywata@den.hokudai.ac.jp; 5 Department of Nutrition, Kagawa Nutrition University, 3-9-21, Chiyoda, Sakado-shi 350-0288, Japan; sshinkai1955@yahoo.co.jp

**Keywords:** eating alone, poor appetite, SNAQ, community-dwelling, nutrition

## Abstract

One prominent factor associated with malnutrition is poor appetite. In Japan, the number of older adults living alone has increased annually. Those living alone tended to eat alone, which may lead to poor appetite. This study aimed to investigate the association between eating alone and poor appetite using an index called the Simplified Nutritional Appetite Questionnaire (SNAQ). We surveyed 818 people aged 70 and over in Takashimadaira, Itabashi-ku, Tokyo, Japan, in 2016. Comparisons were made between two groups, a poor appetite group (*n* = 295) and a good appetite group (*n* = 523), and results indicate that the poor appetite group had a higher rate of eating alone than the good appetite group (38.0% vs. 20. 1%: *p* < 0.001). Multivariable logistic regression (OR; 95%CI) was performed and poor appetite was significantly associated with the Geriatric Depression Scale (GDS) score (1.707; 1.200–2.427), the number of medications (1.061; 1.007–1.118), JST score (0.894; 0.841–0.950), the indication of “very healthy” on a self-rated health scale (0.343; 0.152–0.774), and reports of eating alone (1.751; 1.130–2.712). Our results suggest that eating alone is associated with a poor appetite.

## 1. Introduction

Japan has pioneered an aging society. In 2018, people aged 65 years and older accounted for 28.1% of the population [1]. Both the average life span and healthy life expectancy, which has no limitations to daily living, have been prolonged in Japanese aged citizens. However, the difference between the average life span and healthy life expectancy was about 9 years for men and about 12 years for women, without much change in this difference between 2001 and 2016. It is thought that this is related to the increase in the number of people who qualify for long-term care needs [2]. In Japan, preventive care has been promoted to reduce long-term care needs, prevent deterioration in health, and enhance the quality of life. Preventive care measures include maintaining locomotive function and preventing malnutrition. One of the main factors associated with malnutrition is poor appetite. Poor appetite is common in older people [3], even in those who are healthy and well-nourished. Appetite is often evaluated by whether it is present or whether there is a decrease in dietary intake. The presence of appetite is assessed through simple evaluations of “yes or no” questionnaires. It is difficult to assess changes in one’s own dietary intake. The Simplified Nutritional Appetite Questionnaire (SNAQ) is a tool that quantifies appetite. It was derived from the Council on Nutrition Appetite Questionnaire (CNAQ) [4]. The SNAQ is an appetite assessment tool that predicts weight loss and thus helps detect malnutrition early on and intervene [4]. In other words, it is a periodic assessment used to prevent malnutrition.

In Japan, the number of elderly people living alone has increased annually. Living alone is associated with a higher rate of malnutrition [5] and has been reported to lead to increased long-term care status and mortality [6]. Particularly, an increase in living alone leads to more frequent eating alone. It has been reported that eating alone is associated with low dietary variety and food intake [7,8,9]. Eating alone may also occur among those who live with cohabitants due to difference in life behavior patterns. Previous studies have reported that individuals who eat alone, despite living with others, are more likely to be depressed than their counterparts who eat alone because they live alone [10]. Moreover, depression affects appetite and contributes to malnutrition. Thus, the avoidance of eating alone or encouraging eating with others may help prevent malnutrition. However, no studies have yet investigated whether eating alone is associated with poor appetite.

In this study, we aimed to investigate the association between eating alone and poor appetite using the SNAQ, an appetite index, to objectively assess appetite.

## 2. Materials and Methods

### 2.1. Participants

From July to December 2016, a mail survey and a measurement survey were conducted in Takashimadaira district, Itabashi-ku, Tokyo (this was known as the Takashimadaira Study). The measurement survey was sent to those who responded to the first mail survey, and 1354 subjects participated. Of these, 818 subjects who completed the SNAQ survey and had no deficiency in covariates were included in the final analysis.

This study was conducted with the approval of the Ethical Committee of Tokyo Metropolitan Institute of Gerontology (Nos. 9, 31, 2016).

### 2.2. Appetite Assessment

The SNAQ was developed as a brief assessment tool to predict involuntary weight loss. It comprises four question items (appetite, feeling of satiety, taste, and the number of meals per day) answered on a 5-point scale. The sum of each item in the SNAQ ranges from 4 to 20 points, with lower scores indicating poor appetite. Study participants with the SNAQ scores of ≤14 points were defined as having poor appetite.

### 2.3. Determination of Eating Alone

Participants were asked to answer the question, “How often do you usually eat a meal with others?” by diet category. There were five options: “I usually eat with someone”, “4–5 days a week”, “2–3 days a week”, “1 day a week”, and “I always eat alone”. Those who ate less than three meals a day were excluded, and those who responded “I always eat alone” for all three meals were defined as “eating alone”.

### 2.4. Covariates

The survey participants answered self-administered questionnaires and were interviewed by pretrained nurses and psychologists, and their body composition and physical function were measured by the researchers. The variables examined were gender, age, body composition, subjective olfactory changes, medical history (depression, dementia, gastrointestinal diseases, cardiac diseases, kidney diseases, respiratory disorders, malignant neoplasms, etc.), acute illness within the past three months, drinking habits, smoking status, and the number of medications. Other covariables included the Geriatric Depression Scale 15 (GDS) as a measure of depression, the Mini-Mental State Examination (MMSE) as a measure of cognitive function, the Japan Science and Technology Agency Index of Competence (JST-IC) as a measure of life activity ability, self-reported health, household status (living alone or living together), subjective economic status, social participation at least once a month, and social isolation.

The GDS scores were calculated and divided into two groups by score: <5 as healthy and ≥5 as predisposed to depression. Subjective health and subjective economic conditions were grouped according to significant differences after performing the Kruskal–Wallis test followed by the Bonferroni test. Self-rated health was scored as “not healthy”, “healthy”, and “very healthy”, and subjective economic conditions were scored as “not difficult” or “difficult”. Social participation was reported as “yes” if any activity, such as those related to hobby circles, volunteering, and sports clubs, was pursued at least once a month. Social isolation was reported as “yes” if there was interaction with friends/neighbors or separated family members/relatives fewer than once a week.

### 2.5. Data Analysis

We compared the two categories of the SNAQ scores. Categorical variables were subjected to a chi-square test and continuous variables were subjected to a Mann–Whitney U test presented as the number of people (%) and mean ± SD, respectively. To investigate factors associated with appetite, categories of the SNAQ scores were used as objective variables, and sex, age, and items with significant differences in the comparison between two categories were entered as explanatory variables to perform multivariable logistic regression analyses. Statistical analyses were performed using SPSS Statistics 23 (IBM, Armonk, NY, USA), with a significance level set at *p* < 0.05.

## 3. Results

Based on the SNAQ scores, the participants were divided into two groups: good appetite (>14; 523 participants) and poor appetite (≤14; 295 participants). Comparisons between the two groups showed that the poor appetite group had a higher rate of eating alone than the good appetite group (38.0% vs. 20.1%: *p* < 0.001). In addition, those with poor appetite were more depressed and had higher rates of social disadvantages (Table 1).

Multivariable logistic regression (OR; 95%CI) showed significant associations with GDS (1.707; 1.200–2.427), number of oral medications (1.061; 1.007–1.118), JST score (0.894; 0.841–0.950), “very healthy” self-rated health (0.343; 0.152–0.774), and eating alone (1.751; 1.130–2.712) (Table 2).

## 4. Discussion

Frailty is considered a preliminary step in identifying long-term care status and its prevention. The frailty cycle proposed by Fried et al. [11] is based on the idea that the effects of frailty factors are cyclical. Among these factors, poor appetite has been shown to accelerate the frailty cycle. Therefore, early identification and intervention for poor appetite could interrupt the frailty cycle or slow its acceleration. Previous studies on appetite have shown that poor appetite causes low dietary intake and malnutrition [12] and psychological factors such as depression and well-being are associated with appetite [13]. Early identification of poor appetite and its root causes may help prevent frailty and long-term care status. Furthermore, eating alone has been reported to be associated with frailty [14], indicating reduced nutrient intake [7] and dietary variety [8,9], and thus puts individuals at increased risk of malnutrition. In the dietary cultural backdrop of Japan, eating is considered a communication medium, and sharing meals with others such as family members is preferred [10]. Therefore, eating alone is generally considered pitiable and has a high psychological impact [15]. Certain living conditions among older people, such as low income and food accessibility, are related to eating alone [16]. However, no study has previously reported that eating alone is related to appetite. Although poor appetite is greatly affected by depression and acute symptoms, this study clarifies that eating alone was related to poor appetite even after adjusting for the above factors, suggesting that eating alone affects appetite.

Of the total participants in this study, 38.1% were living alone and 26.5% were eating alone in their households (female—30.8%, male—21.0%). According to the results of this nationwide survey, the ratio of living alone was 26.4%, the ratio of eating alone was 14.2% for women and 11.5% for men—the respective ratios in the case of the participants in this study were higher [17]. In our study area, Tokyo, members of the elderly population living alone comprised the majority. Eating alone may also be attributed to differences in the routines of cohabitants [18]. Remarkably, the employment rate of older people is rising each year across Japan [1] and in Tokyo. Therefore, it is possible that differences in activity times may also occur in households with older inhabitants. Consequently, the participants in this study may have had a higher rate of living alone and eating alone.

Our results clarified that poor appetite was 1.75 times more likely to occur when participants ate alone. In other words, eating alone may lead to poor appetite, which in turn may lead to malnutrition. We defined eating alone as responding to the question “I always eat alone” for every meal in a week. Therefore, eating with others once a week may contribute to improving appetite and preventing malnutrition.

Preventive long-term care projects are being promoted throughout Japan, and it is important not only to provide services to the elderly populations but also to society as a whole, including the surrounding environment. Among these projects, the concept of a “community-based salon for the elderly” has been developed as a place where older people can participate independently rather than being service receivers. Activities in the salon include physical exercise, leisure/hobby activities, eating together, tea time, and activities to prevent dementia. The activity of eating together aims to prevent malnutrition; however, this activity has an implementation rate of less than 5% of the total facilitation of the community-based salon for the elderly. The results of this study suggest that sharing a weekly meal with others reduces the rate of poor appetite and the risk of malnutrition. Previous studies have reported that people who eat with same-sex friends experience increased energy intake [19], and those who frequently eat together are more likely to feel better about themselves and have a wider social network that provides them with social and emotional support [20]. In addition, Sakurai et al. [21] reported that despite living with others, those who eat alone are more prone to depression. Thus, eating with others in a community-based salon for the elderly may offer an opportunity for older adults to eat together outside the household, expand their social network, and further prevent malnutrition and depression.

This study assessed appetite using the SNAQ survey. The SNAQ is a simple and easy-to-use tool that yields results in a short time and can be used by anyone, professional or otherwise. Therefore, it can be implemented in any setting and may encourage the establishment of new services for older people eating together and participating in the community. The community-based salon for the elderly may contribute to the widening of the local community of older adults eating with others and may help prevent malnutrition.

This study has some limitations. This study may not be generalizable because the investigation was conducted in a single urban area. In addition, some survey questions rely on the participants’ own memories and may be affected by the participants’ cognitive function. The MMSE scores of participants were high with an average of 27.2 points; therefore, the effect of cognitive decline is considered to be low. Furthermore, because this was a cross-sectional study, causal association was not clear. In the future, it is necessary to conduct a longitudinal examination.

In summary, eating alone was associated with poor appetite among older adults. Therefore, it is desirable to increase the projects of eating together and participants in the community-based salon for the elderly.

## Figures and Tables

**Table 1:** Comparison between appetite and baseline characteristics.

		Good (>14)	Poor (≤14)	*p*
Sex	Female	301 (57.6)	164 (55.6)	0.607
	Male	222 (42.4)	131 (44.4)	
Age	(years)	76.7 ± 4.8	77.3 ± 4.9	0.075
Height	(cm)	156.0 ± 8.7	156.1 ± 8.9	0.890
Weight	(kg)	56.6 ± 10.1	56.8 ± 11.0	0.898
BMI	(kg/m^2^)	23.2 ± 3.1	23.2 ± 3.6	0.922
Subjective olfactory changes	Unchanged	459 (88.4)	252 (86.3)	0.376
	Dull	60 (11.6)	40 (13.7)	
Depression	No	502 (97.1)	277 (94.5)	0.085
	Yes	15 (2.9)	16 (5.5)	
GDS	(scores)	3.0 ± 3.0	5.2 ± 3.8	**<0.001**
	<5 (healthy)	397 (75.9)	153 (51.9)	**<0.001**
	≥5 (predisposition to depression	126 (24.1)	142 (48.1)	
Dementia	No	516 (99.0)	288 (98.3)	0.345
	Yes	5 (1.0)	5 (1.7)	
MMSE	(scores)	27.4 ± 2.2	26.9 ± 2.8	0.058
Gastrointestinal diseases	No	328 (63.0)	192 (65.8)	0.447
	Yes	193 (37.0)	100 (34.2)	
Heart diseases	No	406 (78.4)	225 (76.8)	0.599
	Yes	112 (21.6)	68 (23.2)	
Kidney diseases	No	475 (91.3)	266 (90.5)	0.702
	Yes	45 (8.7)	28 (9.5)	
Respiratory disorder	No	412(79.5)	227 (78.0)	0.653
	Yes	106 (20.5)	64 (22.0)	
Malignant neoplasms	No	420 (80.9)	248 (85.8)	0.082
	Yes	99 (19.1)	41 (14.2)	
Acute illness (within 3 months)	No	473 (90.4)	242 (82.0)	**<0.001**
	Yes	50 (9.6)	53 (18.0)	
Drinking habits	Does not drink/quit	284 (55.6)	175 (60.1)	0.235
	Drink	227 (44.4)	116 (39.9)	
Smoking habits	Does not smoke/quit	490 (93.7)	269 (91.2)	0.206
	Smoke	33 (6.3)	26 (8.8)	
Medicines	(numbers)	3.4 ± 2.9	4.4 ± 3.6	**<0.001**
JST-IC	(scores)	10.9 ± 2.7	9.3 ± 3.2	**<0.001**
Eating alone	No	418 (79.9)	183 (62.0)	**<0.001**
	Yes	105 (20.1)	112 (38.0)	
Household status	Living together	346 (66.2)	160 (54.2)	**<0.001**
	Living alone	177 (33.8)	135 (45.8)	
Self-rated health	Not healthy	69 (13.2)	77 (26.1)	**<0.001**
	Healthy	381 (72.8)	208 (70.5)	
	Very healthy	73 (14.0)	10 (3.4)	
Subjective economic conditions	Not difficult	90 (17.2)	28 (9.5)	**0.003**
	Difficult	433 (82.8)	267 (90.5)	
Social participation	Yes	383 (73.2)	194 (65.8)	**0.026**
	No	140 (26.8)	101 (34.2)	
Social isolation	No	385 (73.6)	190 (64.4)	**0.007**
	Yes	138 (26.4)	105 (35.6)	

Chi-square test: *N* (%)/Mann–Whitney U test: mean ± SD. BMI: body mass index, GDS: Geriatric Depression Scale 15, MMSE: Mini-Mental State Examination, JST-IC: Japan Science and Technology Agency Index of Competence. Bold indicates a *p*-value of less than 0.05.

**Table 2:** Odds ratios and 95% confidence intervals for eating alone according to appetite groups.

		OR	(95% CI)	*p*-Value
Sex	(0: female, 1: male)	1.145	(0.815–1.609)	0.435
Age	(years; per 1-year)	0.991	(0.958–1.024)	0.576
GDS	(0: healthy, 1: predisposition to depression)	1.707	(1.200–2.427)	**0.003**
Acute illness	(0: no, 1: yes)	1.534	(0.950–2.475)	0.080
Medicines	(numbers; per 1-number)	1.061	(1.007–1.118)	**0.026**
JST-IC	(scores; per 1-score)	0.894	(0.841–0.950)	**<0.001**
Household status	(0: together, 1: alone)	1.058	(0.696–1.608)	0.792
Self-rated health	(0: not healthy)	Ref.		
	(1: healthy)	0.940	(0.607–1.455)	0.781
	(2: very healthy)	0.343	(0.152–0.774)	**0.010**
Subjective economic conditions	(0: not difficult, 1: difficult)	1.554	(0.961–2.514)	0.072
Social participation	(0: yes, 1: no)	0.860	(0.598–1.237)	0.417
Social isolation	(0: no, 1: yes)	1.136	(0.786–1.641)	0.497
Eating alone	(0: no, 1: yes)	1.751	(1.130–2.712)	**0.012**

Dependent variable: SNAQ groups (0: good (>14), 1: poor (≤14)). OR: odds ratio, CI: confidence interval, GDS: Geriatric Depression Scale 15, JST-IC: Japan Science and Technology Agency Index of Competence. Bold indicates a *p*-value of less than 0.05.
